# Metabolomics detects clinically silent neuroinflammatory lesions earlier than neurofilament-light chain in a focal multiple sclerosis animal model

**DOI:** 10.1186/s12974-022-02614-8

**Published:** 2022-10-09

**Authors:** Tianrong Yeo, Halwan Bayuangga, Marcus Augusto-Oliveira, Megan Sealey, Timothy D. W. Claridge, Rachel Tanner, David Leppert, Jacqueline Palace, Jens Kuhle, Fay Probert, Daniel C. Anthony

**Affiliations:** 1 Department of Pharmacology, University of Oxford, Mansfield Road, Oxford, OX1 3QT UK; 2 Department of Neurology, National Neuroscience Institute, Singapore, Singapore; 3 Duke-NUS Medical School, Singapore, Singapore; 4 Lee Kong Chian School of Medicine, Nanyang Technological University, Singapore, Singapore; 5 Nuffield Department of Clinical Neurosciences, University of Oxford, Oxford, UK; 6 Department of Neurology, Faculty of Medicine, Public Health, and Nursing, Gadjah Mada University, Yogyakarta, Indonesia; 7 Laboratório de Farmacologia Molecular, Instituto de Ciências Biológicas, Universidade Federal Do Pará, Belém, Brazil; 8 Programa de Pós-Graduação em Farmacologia e Bioquímica, Instituto de Ciências Biológicas, Universidade Federal do Pará, Belém, Brazil; 9 Department of Chemistry, Chemistry Research Laboratory, University of Oxford, Mansfield Road, Oxford, OX1 3TA UK; 10 The Jenner Institute, University of Oxford, Oxford, UK; 11 Department of Biology, University of Oxford, Oxford, UK; 12 Departments of Medicine, Biomedicine and Clinical Research, University Hospital Basel and University of Basel, Neurologic Clinic and Policlinic, Basel, Switzerland; 13 Department of Neurology, Oxford University Hospitals Trust, Oxford, UK

**Keywords:** Multiple sclerosis, Metabolomics, Neurofilament-light, Delayed-type hypersensitivity, Biomarker, Blood, Cerebrospinal fluid

## Abstract

**Background:**

Despite widespread searches, there are currently no validated biofluid markers for the detection of subclinical neuroinflammation in multiple sclerosis (MS). The dynamic nature of human metabolism in response to changes in homeostasis, as measured by metabolomics, may allow early identification of clinically silent neuroinflammation. Using the delayed-type hypersensitivity (DTH) MS rat model, we investigated the serum and cerebrospinal fluid (CSF) metabolomics profiles and neurofilament-light chain (NfL) levels, as a putative marker of neuroaxonal damage, arising from focal, clinically silent neuroinflammatory brain lesions and their discriminatory abilities to distinguish DTH animals from controls.

**Methods:**

^1^H nuclear magnetic resonance (NMR) spectroscopy metabolomics and NfL measurements were performed on serum and CSF at days 12, 28 and 60 after DTH lesion initiation. Supervised multivariate analyses were used to determine metabolomics differences between DTH animals and controls. Immunohistochemistry was used to assess the extent of neuroinflammation and tissue damage.

**Results:**

Serum and CSF metabolomics perturbations were detectable in DTH animals (vs. controls) at all time points, with the greatest change occurring at the earliest time point (day 12) when the neuroinflammatory response was most intense (mean predictive accuracy [SD]—serum: 80.6 [10.7]%, *p* < 0.0001; CSF: 69.3 [13.5]%, *p* < 0.0001). The top discriminatory metabolites at day 12 (serum: allantoin, cytidine; CSF: glutamine, glucose) were all reduced in DTH animals compared to controls, and correlated with histological markers of neuroinflammation, particularly astrogliosis (Pearson coefficient, *r*—allantoin: *r* = − 0.562, *p* = 0.004; glutamine: *r* = − 0.528, *p* = 0.008). Serum and CSF NfL levels did not distinguish DTH animals from controls at day 12, rather, significant differences were observed at day 28 (mean [SEM]—serum: 38.5 [4.8] vs. 17.4 [2.6] pg/mL, *p* = 0.002; CSF: 1312.0 [379.1] vs. 475.8 [74.7] pg/mL, *p* = 0.027). Neither serum nor CSF NfL levels correlated with markers of neuroinflammation; serum NfL did, however, correlate strongly with axonal loss (*r* = 0.641, *p* = 0.001), but CSF NfL did not (*p* = 0.137).

**Conclusions:**

While NfL levels were elevated later in the pathogenesis of the DTH lesion, serum and CSF metabolomics were able to detect early, clinically silent neuroinflammation and are likely to present sensitive biomarkers for the assessment of subclinical disease activity in patients.

**Supplementary Information:**

The online version contains supplementary material available at 10.1186/s12974-022-02614-8.

## Introduction

At least 90% of multiple sclerosis (MS) lesions detectable by magnetic resonance imaging (MRI) are asymptomatic as these involve clinically ineloquent areas [1]. Although MRI is invaluable in identifying subclinical lesions, it is performed infrequently, thus, it is difficult to determine the time at which a new subclinical lesion appears. Hence, there is great interest in biofluid markers to enable the frequent monitoring of subclinical disease activity. While none are currently used in routine clinical practice, neurofilament-light chain (NfL), a marker of neuroaxonal damage [2], appears promising as it is associated with several indices of subclinical disease activity including baseline T_2_ and gadolinium-enhancing lesions [3, 4], prospective new/enlarging T_2_ lesions [3], and decreases after initiation of disease-modifying therapies [3–6].

Metabolomics is a downstream ‘omics’ approach involving the comprehensive study of low molecular weight metabolites and has emerged as a useful tool for biomarker discovery and unravelling perturbed metabolic pathways in diseases [7]. Using this approach in MS patients, we have shown that beyond diagnostic purposes [8–11], metabolomics can offer useful information on clinical activity by distinguishing relapse from remission at both group and individual levels [12], and predicting the clinical conversion of clinically isolated syndrome to MS [13].

Given the dynamic nature of metabolic perturbations generated by immune-mediated pathology, it is feasible that the evaluation of the metabolome might enable the early detection of biochemical changes arising from clinically silent brain lesions as they develop. As patients are already symptomatic at presentation, often with evidence of prior subclinical lesions, animal models provide a means to better understand the local and systemic metabolic alterations resulting from neuroinflammatory lesions independent of their functional sequelae. Indeed, to understand whether it is even possible to detect an ‘echo’ of a single MS-like lesion in the periphery and at what stage it might become possible to detect this, is an important matter that needs to be addressed in order to shed light on the nature and source of the metabolic (and indeed NfL) changes that have been observed in patients. Metabolomics studies using experimental autoimmune encephalomyelitis (EAE) models have shown high accuracy in distinguishing EAE animals from non-immunized and naïve controls [14–20], however, several features make EAE less ideal to study metabolic changes resulting from neuroinflammation. Firstly, EAE lesions are random with regard to their topography and size—an important consideration as different brain regions have varying metabolic make up [21], hence, this is likely to generate inconsistent metabolic profiles. Secondly, EAE models are associated with overt clinical signs such as weight loss and paralysis that are disproportionate to human MS [22], thus, these studies are unable to disentangle the contribution of neuroinflammatory lesions from their functional sequelae to the metabolic perturbations observed.

An animal model producing a focal lesion in a pre-determined locale within the central nervous system (CNS) without overt clinical signs would hence be more suitable. The delayed-type hypersensitivity (DTH) MS model, first described by Matyszak and Perry almost 30 years ago [23], produces a focal, cell-mediated neuroinflammatory lesion consisting of T-cells, macrophages, reactive microglia and astrocytes, with demyelination and axonal loss [23–25]. The acute DTH lesion is considered to be fully established between days 12–19 after peripheral sensitization, however, chronic inflammation persists and blood–brain barrier breakdown is still present albeit less marked at day 31 [24, 25]. Of note, the DTH model does not produce any observable motor or behavioural disturbances [26], eradicating the concern of aberrant metabolic contributions from weight loss, paralysis, and reduced food/water intake.

In this study, using the DTH model, we sought to: (1) investigate the temporal dynamics of metabolomics profiles and NfL concentrations in the serum and cerebrospinal fluid (CSF) throughout the evolution of the DTH lesion, (2) identify the principal discriminatory serum and CSF metabolites distinguishing DTH from control animals, and (3) correlate these metabolites and NfL to histological markers of neuroinflammation and myelin and axonal damage.

## Materials and methods

**Fig. 1:**
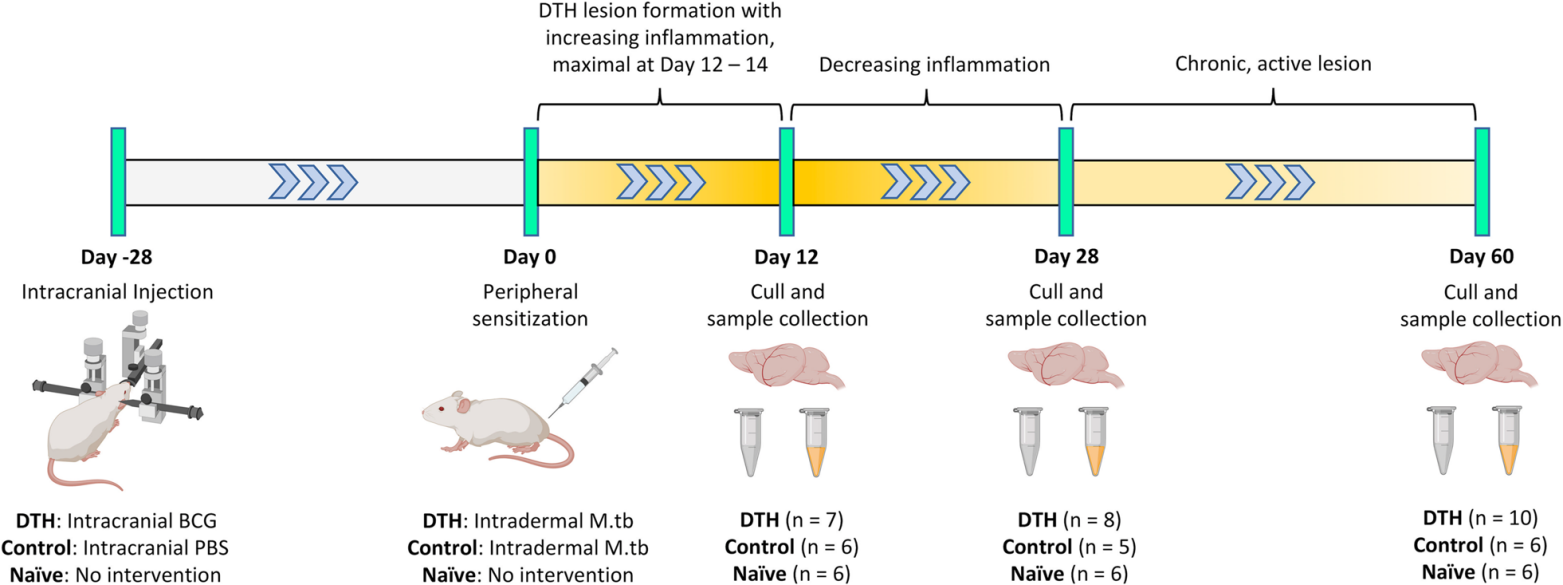
Overview of the different experimental groups and time points in the study. *BCG* Bacillus Calmette–Guérin, *DTH* delayed-type hypersensitivity, *M.tb* *Mycobacterium tuberculosis*, *PBS* phosphate-buffered saline

An overview of the experimental groups and time points is shown in Fig. 1.

### Induction of the DTH lesion

Four-week-old male wild-type Lewis rats (Charles River UK) were obtained and randomly assigned to DTH, control, and naïve groups.

Following isoflurane-induced anaesthesia, the head of the animals were shaved, sterilized and mounted on a stereotaxic frame. Under an operating microscope, a small midline scalp incision was made after bupivacaine injection and a burr hole was created using a dental drill at 1 mm anterior and 3 mm lateral to the bregma. Two microlitres of heat-killed Bacillus Calmette–Guérin (BCG) (concentration = 1 × 10^5^ colony forming units (CFU)/μL in sterile phosphate-buffered saline [PBS]) was then injected slowly over 5 min via a finely drawn glass microcapillary into the left striatum 4 mm from the cortical surface, before suturing the incision. For control animals, 2 μL of sterile PBS was injected. No stereotaxic surgery was performed for naïve animals.

Twenty-eight days later, when the initial innate immune response to intracranial BCG has subsided [23], peripheral sensitization was performed to generate the DTH lesion at the site of BCG implantation. An emulsion of Complete Freud’s Adjuvant (Sigma-Aldrich) augmented with additional heat-killed Mycobacterium tuberculosis (M.tb) (BD Difco™ H37Ra) was prepared (final M.tb concentration = 3.5 mg/mL). Under isoflurane-induced anaesthesia, DTH and control animals received 50 μL intradermal injection of the emulsion into each hind limb (i.e. 0.35 mg of M.tb in 100 μL per animal). Naïve animals did not receive any peripheral challenge. Wounds were taken care of as appropriate after all procedures and all animals were housed in standard 12:12 diurnal/nocturnal light cycles with ad libitum access to food and water.

### Tissue sample collection

On days 12, 28, and 60 after peripheral sensitization, animals were anaesthetized (isoflurane-induced), shaved, and mounted with neck hyperflexion to allow for maximal access to the cisterna magna, via which ~ 150 μL of clean CSF was drawn using a 25 G needle attached to a 1 mL syringe. CSF obtained was centrifuged (15,000 g, 10 min, room temperature), aliquoted, and stored immediately at − 80 °C. Following CSF sampling, blood was collected via cardiac puncture, transferred to a serum tube (BD™ 367837), left to stand for 30 min, and then centrifuged (1300 g, 10 min, room temperature). Serum obtained was aliquoted and stored immediately at − 80 °C.

Animals were then transcardially perfused with heparinized saline followed by 4% paraformaldehyde. Brains were harvested, post-fixed in 4% paraformaldehyde overnight and cryoprotected with 30% sucrose, after which they were embedded in OCT compound, frozen in cold isopentane, and stored at − 20 °C.

### Immunohistochemistry

Brains were cryosectioned at 10 μm thickness (Leica Microsystems). Following heat-induced antigen retrieval, quenching and blocking with 10% goat serum, sections were incubated with primary antibodies overnight at 4 °C. The primary antibodies used were: anti-IBA1 (1:2000, Abcam, ab178846) for microglia/macrophages; anti-GFAP (1:2000, Dako, Z0334) for astrocytes; anti-MBP (1:1000, Abcam, ab40390) for myelin, and anti-NfH (1:1000, Abcam, ab8135) for axons.

Sections were then incubated with goat anti-rabbit biotinylated secondary antibody (Vectorlabs) for 2 h, followed by Vectastain^®^ Elite ABC-HRP (Vectorlabs) for 1 h. Antibody binding was visualized using 3, 3′ diaminobenzidine (Sigma-Aldrich) as the chromogen. Sections were then counterstained in cresyl violet, dehydrated, cleared, and mounted with DPX mounting media.

### Histological assessment

Histological assessment was performed by an assessor blinded to experimental groups and time points.

Neuroinflammation was assessed by quantifying the number of IBA1 and GFAP-positive cells in 5 non-overlapping fields at 20X magnification at the medial edge of the outer active border. Images were imported into ImageJ and cells manually counted using the ‘Cell Counter’ function [27]. Final cell density was expressed as mean number of cells/mm^2^ (averaged across the 5 fields).

Tissue damage was quantified by the area within the striatum that was devoid of MBP and NfH staining. Images were obtained using a whole slide image scanner connected to a light microscope at 20X magnification and the area calculated using ImageJ [27], expressed in mm^2^.

### Metabolomics

^1^H nuclear magnetic resonance (NMR) spectroscopy metabolomics was performed using a 700-MHz Bruker AVIII spectrometer operating at 16.4 T equipped with a ^1^H [^13^C/^15^ N] TCI cryoprobe. After thawing, 245 μL of serum or 80 μL of CSF were buffered with 75 mM sodium phosphate buffer, giving a final volume of 550 μL for each NMR sample, which was then transferred to a 5-mm borosilicate glass tube (Norell™).

Serum spectra were acquired using a spin-echo Carr–Purcell–Meiboom–Gill (CPMG) sequence (τ interval of 400 μs, 80 loops, 40 ms total filter time, 32 data collections, 1.5 s acquisition time, relaxation delay of 2 s, fixed receiver gain) to suppress broad signals arising from large molecular weight serum components. CSF spectra were acquired using a 1D-nuclear overhauser effect spectroscopy (NOESY) presaturation scheme (2 s presaturation, 64 data collections, 1.5 s acquisition time, fixed receiver gain) for attenuation of the water resonance. Details of NMR spectra processing have been previously published [10]. Following this, integral values of individual spectral ‘bins’ were computed with constant-sum-normalization and used as quantitative variables expressed in arbitrary units (AU). In total, 194 serum and 122 CSF metabolite ‘bins’ were available for multivariate statistical analysis. Metabolite assignment was done by referencing to literature values and inspection of the 2D-total correlation spectroscopy spectra.

### NfL measurements

CSF and serum NfL levels were determined using the Simoa^®^ assay (Quanterix). Assay methodology has been previously described [28]. Laboratory personnel were blinded to experimental groups and time points.

### Multivariate statistical analysis

Orthogonal partial-least square discriminant analysis (OPLS-DA), using in-house R scripts and the *ropls* package [9, 29, 30], was applied to metabolomics spectral data to construct discriminatory models separating DTH and control animals. All OPLS-DA models were validated on independent test data using *k*-fold external cross-validation with 100 repetitions (*k* = number of animals in the smaller group). This involves repeated cycles of (1) balancing group sizes, (2) random partitioning of the spectral data into a training set and a test set, (3) constructing OPLS-DA models using the training set alone with leave-one-out internal cross-validation, and then (4) determining the predictive accuracy using the independent test set. The metabolomics distinction between animal groups was only valid if the mean predictive accuracy of the ensemble of model accuracies was significantly higher than the mean predictive accuracy of a separate ensemble created by random class assignments (i.e. null distribution) on the same spectral data.

### Univariate statistical analysis

Univariate analysis was performed using GraphPad Prism (La Jolla, CA, USA). Comparative analyses between groups were performed using one-way ANOVA with Tukey post hoc corrections. Two-way ANOVA was used to compare across treatment groups and time points, with Sidak test for multiple comparison corrections. Pearson coefficient, *r*, was used to determine the strength of correlations. Two-tailed *p* values of < 0.05 were considered statistically significant.

## Results

### Characterization of the DTH model

**Fig. 2:**
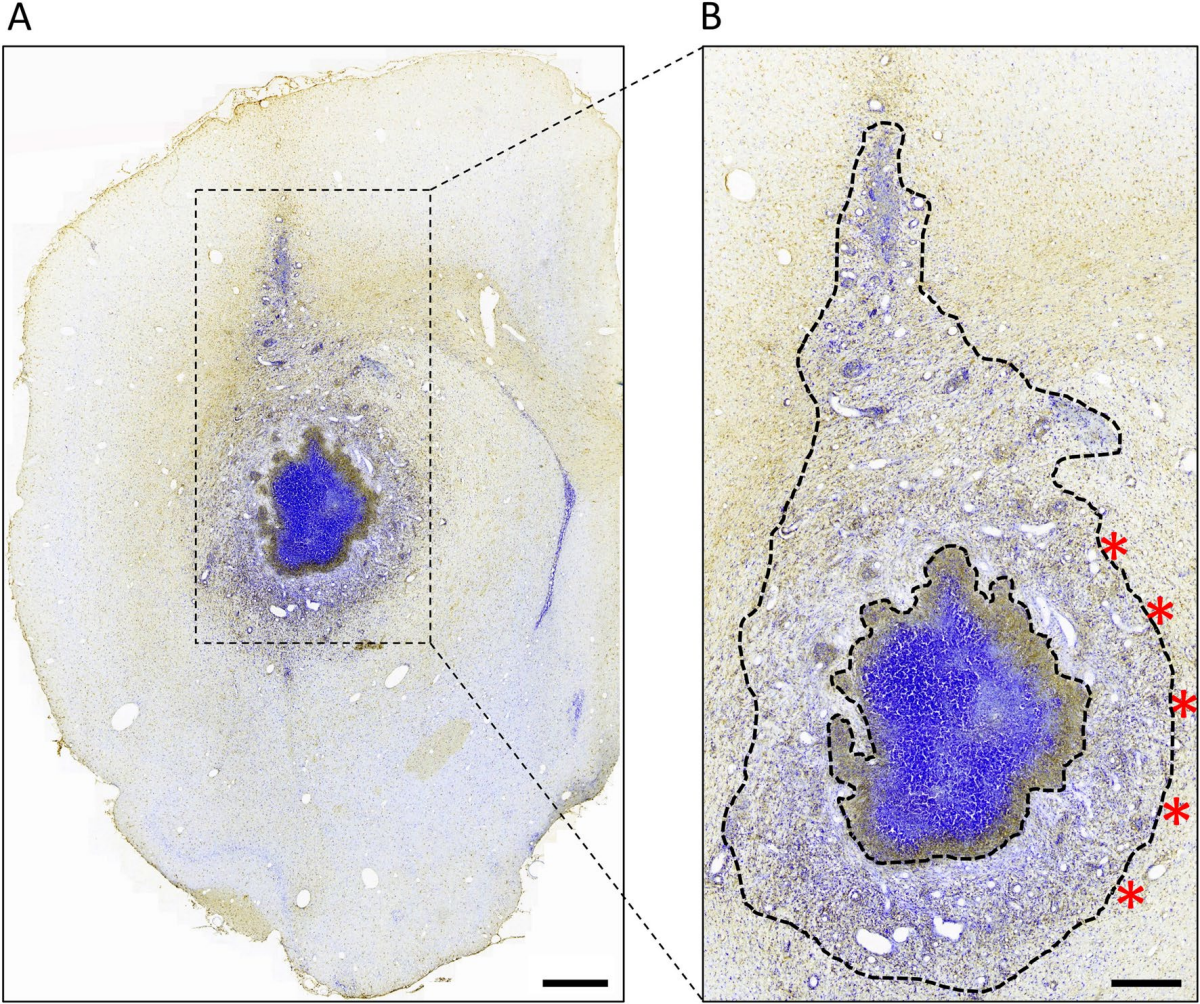
The focal DTH lesion. **A** Day 12 DTH lesion in the left striatum, stained for IBA1 (microglia/macrophages) with cresyl violet counter-staining. Scale bar = 200 μm. **B** On higher magnification, the lesion composed of a hypercellular core (within the inner black dashed line) and an outer active border (between the inner and outer black dashed lines). This outer active border consisted of a dense inflammatory infiltrate, which demonstrated a gradient of decreasing cellularity with increasing distance away from the lesion core. Perivascular cuffing and dilatation of vessels were also observed in this outer active border. Five non-overlapping representative fields (indicated by the red asterisks) at the medial edge of this outer active border were selected for IBA1 and GFAP-positive cell density quantification. Scale bar = 100 μm. *DTH* delayed-type hypersensitivity, *GFAP* glial fibrillary acidic protein, *IBA1* ionized calcium binding adaptor molecule 1

A focal neuroinflammatory lesion centred at the site of BCG implantation was observed in all DTH animals (Fig. 2A). The 5 non-overlapping fields for cell density quantification were taken from the medial edge of the outer active border (Fig. 2B). No lesion was found in the contralateral hemisphere in DTH animals or in control and naïve animals. There were no observable motor disturbances seen in DTH animals, consistent with previous reports [26]—the tail was rigid with no gait disturbances at all times. There were also no weight differences between DTH, control, and naïve animals at each time point (Additional file 1: Fig. S1).

### Histology assessment of neuroinflammation

**Fig. 3:**
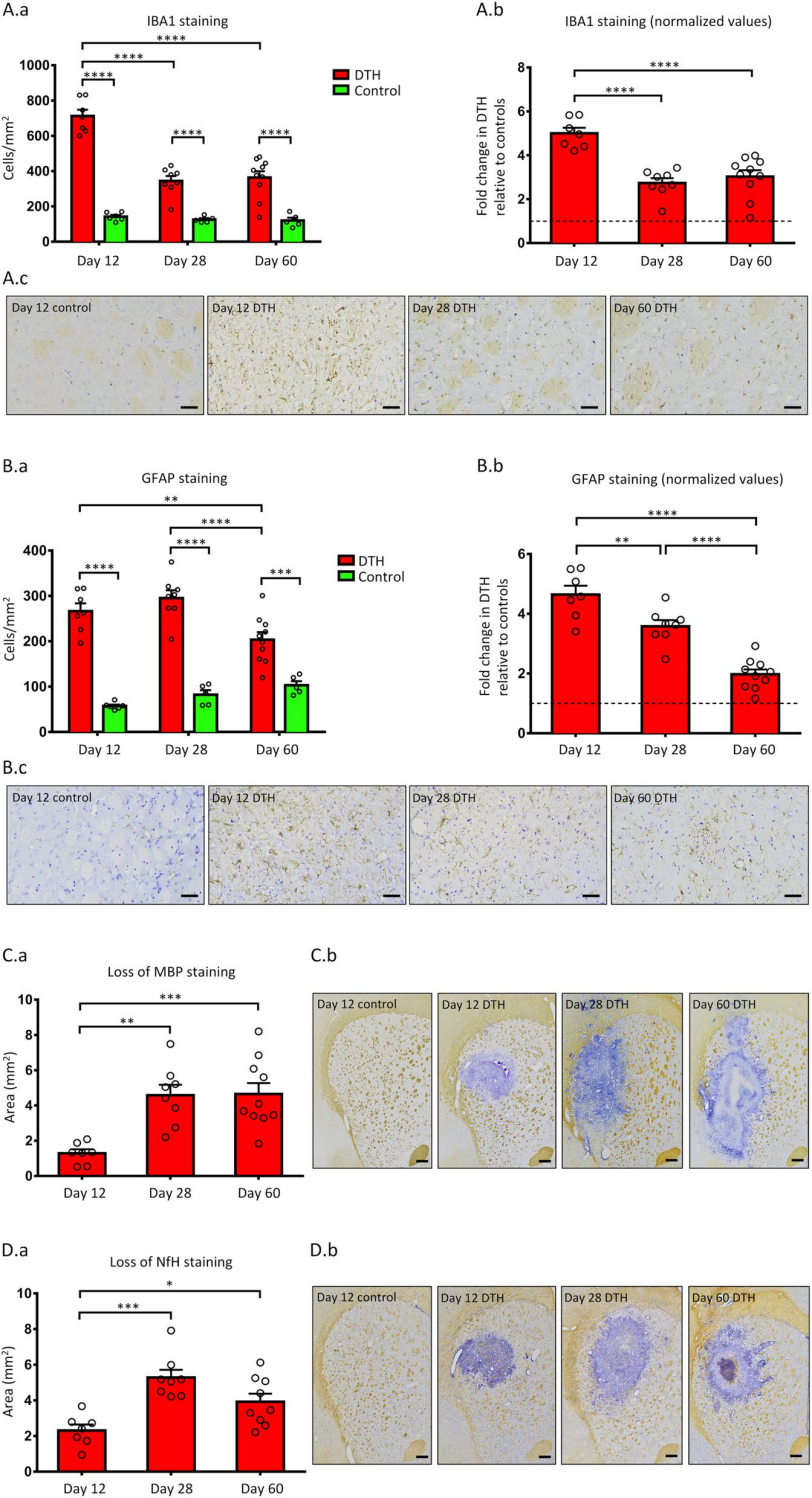
Histological assessment of neuroinflammation and tissue damage. **A** IBA1-positive cell densities in DTH and control animals. The mean density of IBA1-positive cells (microglia/macrophages) was greater in DTH animals compared to controls at all time points, with the peak density noted at day 12 (**a**). Fold change of IBA1-positive cell density within DTH animals, normalized to the mean density of the controls at each time point (indicated by the horizontal dashed line at 1.0), showing that the greatest fold change occurred at day 12 (**b**). Representative images of IBA1 staining in day 12 control and in DTH animals at all 3 time points (**c**). **B** GFAP-positive cell densities in DTH and control animals. The mean density of GFAP-positive cells (astrocytes) was higher in DTH animals compared to controls at all time points (**a**). Fold change of GFAP-positive cell density within DTH animals, showing that the highest fold change was at day 12 and decreased with time (**b**). Representative images of GFAP staining in day 12 control and in DTH animals at the 3 time points (**c**). **C** Area of MBP loss within DTH animals. MBP (myelin) loss was the least pronounced at day 12, increased at day 28, and plateaued at day 60 (**a**). Representative images of MBP staining in day 12 control and in DTH animals at all time points (**b**). **D** Area of NfH loss within DTH animals. NfH (axonal) loss in DTH animals was the lowest at day 12, and increased at days 28 and 60 (**a**). Representative images of NfH staining in day 12 control and in DTH animals at all time points (**b**). ****p < 0.0001, ***p < 0.001, **p < 0.01, and *p < 0.05 by Sidak post hoc test after two-way ANOVA (**A.a** and **B.a**) and by Tukey post hoc test after one-way ANOVA (**A.b**, **B.b**, **C.a**, and **D.a**). All scale bars = 50 μm. Data presented as mean ± SEM. *ANOVA* analysis of variance, *DTH* delayed-type hypersensitivity, *GFAP* glial fibrillary acidic protein, *IBA1* ionized calcium binding adaptor molecule 1, *MBP* myelin basic protein, *NfH* neurofilament-heavy chain, *SEM* standard error of mean

DTH animals had a higher density of IBA1-positive cells (i.e. microglia/macrophages) compared to control animals at all time points (Fig. 3A), with the highest density observed at day 12. To compare microglia/macrophage density within DTH animals while accounting for the basal levels within controls, data in DTH animals were normalized to the mean cell density of controls at each time point. Expectedly, the highest fold change was observed at day 12 with no difference between days 28 and 60. These observations suggest that microglia/macrophage recruitment was most intense at day 12, compatible with previous descriptions [23].

The density of GFAP-positive cells (i.e. astrocytes) was also higher in DTH animals compared to controls at all time points (Fig. 3B). Cell densities of DTH animals were then normalized to the mean of controls at each time point, revealing that within DTH animals, astrocytic density was the highest at day 12 and decreased with time.

### Histology assessment of tissue damage

The area devoid of MBP staining (i.e. myelin loss) within DTH animals was the lowest at day 12, rose at day 28, and plateaued at day 60 (Fig. 3C). Similarly, the area devoid of NfH staining (i.e. axonal loss) was the lowest at day 12, and increased at days 28 and 60 (Fig. 3D). There was no evidence of demyelination or axonal loss in control animals, therefore no normalization was required.

### CSF and serum NfL concentrations across experimental time points

No differences in CSF and serum NfL concentrations were noted between control and naïve animals at any time point (Additional file 2: Fig. S2), consistent with histological observation that intracranial PBS injection followed by peripheral sensitization did not induce axonal injury.

**Fig. 4:**
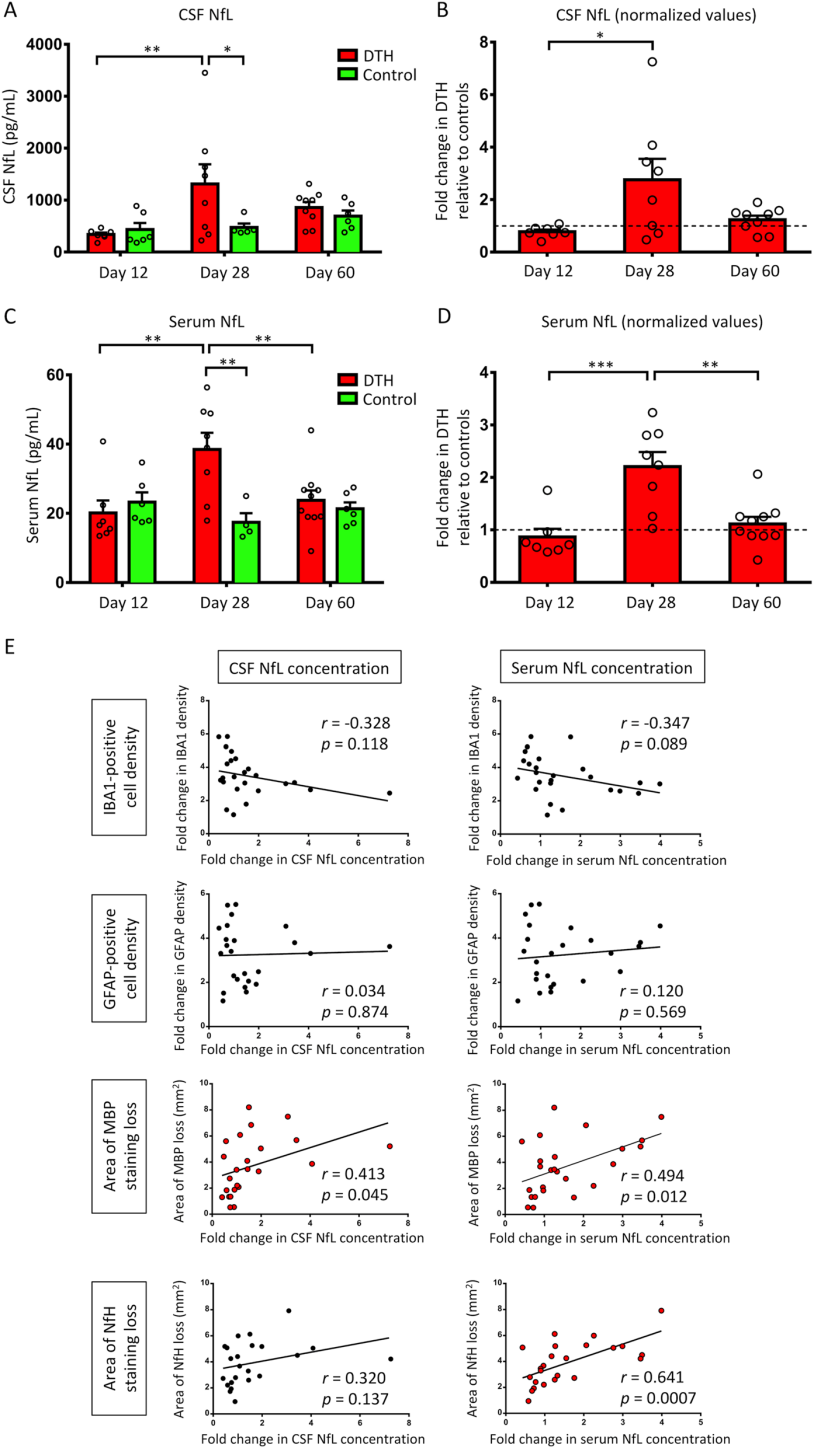
CSF and serum NfL concentrations. **A** CSF NfL concentrations in DTH and control animals. CSF NfL was able to differentiate the 2 groups at day 28, but not at day 12 although there was axonal loss histologically at this time point. **B** Normalized data in DTH with respect to controls (control means at each time point are indicated by the horizontal dashed line at 1.0) revealed that peak CSF NfL concentration was observed at day 28, although the post hoc pair-wise comparison between days 28 and 60 was not statistically significant. **C** Serum NfL concentrations in DTH and control animals. A similar observation was noted for serum NfL whereby the distinction between the 2 groups was made at day 28. **D** Normalized data showing that serum NfL peaked at day 28. **E** Correlation matrix between CSF and serum NfL with histological markers. Statistically significant correlations are indicated by red symbols. ***p < 0.001, **p < 0.01, *p < 0.05 by Sidak post hoc test after two-way ANOVA (**A** and **C**) and by Tukey post hoc test after one-way ANOVA (**B** and **D**). Data presented as mean ± SEM. *ANOVA* analysis of variance, *DTH* delayed-type hypersensitivity, *GFAP* glial fibrillary acidic protein, *IBA1* ionized calcium binding adaptor molecule 1, *MBP* myelin basic protein, *NfH* neurofilament-heavy chain, *NfL* neurofilament-light chain, *r* Pearson correlation coefficient, *SEM* standard error of mean

At day 12, CSF and serum NfL levels were not higher in DTH animals compared to controls. However, a significant increase was observed at day 28 (mean [SEM]; CSF NfL: 1312.0 [379.1] vs. 475.8 [74.7] pg/mL, *p* = 0.027; serum NfL: 38.5 [4.8] vs. 17.4 [2.6] pg/mL, *p* = 0.002), which resolved by day 60 (Fig. 4A, C). NfL concentrations in DTH animals were then normalized to the mean concentrations of controls at each time point to correct for basal levels. This revealed that NfL levels increased and peaked at day 28, with a subsequent decrease at day 60 although the decline in CSF NfL was not statistically significant between days 28 and 60 (Fig. 4B, D).

### Correlation of CSF and serum NfL with histology

Correlations of CSF and serum NfL with histological markers were performed to determine if NfL can represent a surrogate marker for neuroinflammation and tissue damage throughout the DTH disease course. Normalized data for NfL levels, IBA1 and GFAP-positive cell densities were used for correlation analyses to account for basal levels within controls, thus representing the actual contribution by the DTH lesion. As there was no myelin or axonal loss observed in controls, absolute values in DTH animals were used. A moderate correlation was observed for serum NfL concentration with myelin loss (*r* = 0.494, *p* = 0.012), with a weaker correlation for CSF NfL (*r* = 0.413, *p* = 0.045) (Fig. 4E). There was a moderately strong correlation for serum NfL concentration with histologically determined axonal loss (NfH staining) (*r* = 0.641, *p* = 0.0007) but not for CSF NfL (*p* = 0.137).

### Correlation of CSF and serum NfL levels

It is argued that following axonal injury, NfL is first released into the extracellular space, then into CSF, and finally into blood [31]. Clinical studies in various neurological diseases have reported moderate to strong correlations of CSF and blood NfL concentrations (*r* range: 0.53–0.89) [4, 5, 32, 33], hence, it was explored if such a correlation was also present in the DTH model.

Within all animals across all time points, a moderately strong correlation was observed (*r* = 0.642, *p* < 0.0001), as was the case within DTH animals (*r* = 0.666, *p* = 0.0005) (Additional file 3: Fig. S3). However, no correlations were noted within control or naïve animals, and when DTH animals were stratified by time points, moderately strong correlations were observed at days 28 (*r* = 0.698) and 60 (*r* = 0.654), but not at day 12.

### Serum metabolomics across experimental time points

To identify if serum metabolomics perturbations are present due to the presence of the DTH lesion, OPLS-DA was used to construct discriminatory models on CPMG spectral data to distinguish DTH and control animals.

**Fig. 5:**
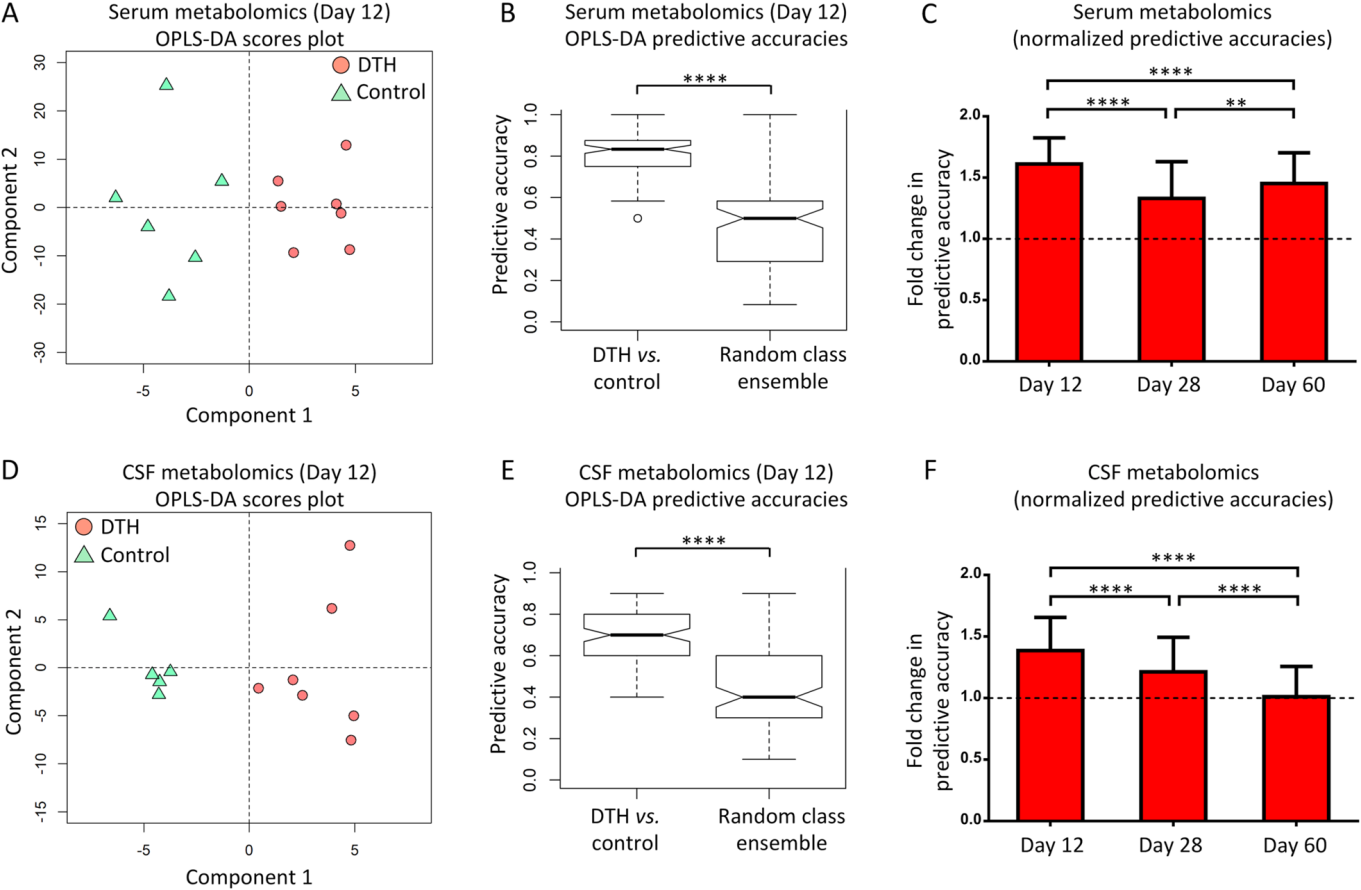
Serum and CSF metabolomics. **A** Representative scores plot derived from OPLS-DA modelling on serum CPMG spectral data of DTH and control animals. **B** Box plots of predictive accuracies from the serum OPLS-DA models of DTH vs. control animals ensemble, against random class ensemble. **C** Fold change in predictive accuracies of the serum OPLS-DA models of DTH vs. controls at each time point, normalized to random chance (indicated by the horizontal dashed line at 1.0), showing that the greatest serum metabolomics perturbation occurred at day 12. **D** Representative scores plot derived from OPLS-DA modelling on CSF NOESY-presat spectral data. **E** Predictive accuracies from the CSF OPLS-DA models of DTH vs. control animals, against random class assignment. **F** Fold change in predictive accuracies of the CSF OPLS-DA models of DTH vs. controls at each time point (horizontal dashed line at 1.0 indicate random chance), revealing that maximal CSF metabolomics perturbation occurred at day 12. ****p < 0.0001 by Kolmogorov–Smirnov test (**B** and **E**). ****p < 0.0001, **p < 0.01 by post hoc Tukey test after one-way ANOVA (**C** and **F**). Data presented as mean ± SD in **C** and **F**. *ANOVA* analysis of variance, *CPMG* Carr–Purcell–Meiboom–Gill, *DTH* delayed-type hypersensitivity, *NOESY* nuclear overhauser effect spectroscopy, *OPLS-DA* orthogonal partial-least square discriminant analysis, *SD* standard deviation

At day 12, the representative OPLS-DA scores plot showed a clear separation between DTH and control animals (Fig. 5A). It is worth noting that while the scores plot enables visualization of the OPLS-DA model output, it is representative and do not illustrate the full validation procedure that was applied (i.e. robust external cross-validation with permutation testing); it is the predictive accuracy from the cross-validation that should be interpreted. The mean predictive accuracy for the ensemble of the OPLS-DA models comparing DTH vs. control animals was significantly higher than the mean predictive accuracy of the ensemble created by random class assignments (mean [SD]; 80.6 [10.7]% vs. 45.9 [20.6]%, *p* < 0.0001) (Fig. 5B), validating the serum metabolomics differences between the 2 groups.

The predictive accuracy for the ensemble of the OPLS-DA models for DTH vs. control animals at day 28 was significantly higher than that of the random class ensemble (mean [SD]; 66.6 [14.9]% vs. 45.7 [19.1]%, *p* < 0.0001), while this was 72.7 (12.5)% vs. 44.4 (17.7)% (*p* < 0.0001) at day 60, confirming the presence of serum metabolomics differences at these time points.

The fold changes in the predictive accuracies (normalized to the accuracy of random chance, i.e. 50%) of DTH vs. control animals at each time point are shown in Fig. 5C, highlighting that the maximal serum metabolomics perturbation within DTH animals (vs. controls) occurred at day 12 when there is greatest neuroinflammatory intensity.

### CSF metabolomics across experimental time points

A similar analytical strategy was employed to explore if CSF metabolomics alterations were also present at different time points. CSF samples with visible blood contamination were excluded from metabolomics analysis as this can introduce aberrant NMR resonances in the CSF spectra (Additional file 4: Fig. S4). There were no CSF samples with aberrant NMR resonances that had not already been noted by visual inspection for blood contamination during CSF collection. Indeed, only 1 CSF sample (a day 12 control) had to be excluded.

OPLS-DA models were constructed using NOESY-presat spectral data for DTH vs. control animals at day 12, with the representative scores plot revealing a distinct separation between the 2 groups (Fig. 5D). The mean predictive accuracy for the ensemble of the OPLS-DA models of DTH vs. control animals was significantly higher than that of the random class ensemble (mean [SD]; 69.3 [13.5]% vs. 42.9 [19.3]%, *p* < 0.0001) (Fig. 5E), validating the CSF metabolomics differences.

For day 28, the predictive accuracy for the DTH vs. controls ensemble of OPLS-DA models was higher than that of the random ensemble (mean [SD]; 60.7 [14.0]% vs. 44.0 [20.8]%, *p* < 0.0001), while the mean accuracy at day 60 was 50.6 (12.3)% for the defined-class ensemble against 44.3 (18.4)% for the random ensemble (*p* = 0.0008). These results suggest that although there were still CSF metabolomics differences between DTH and control animals at these latter time points, they were less pronounced than those observed at day 12 as illustrated in Fig. 5F, showing that the fold changes in predictive accuracies (normalized to accuracy of random chance, i.e. 50%) decreased over time.

### Top discriminatory serum and CSF metabolites

To identify the most discriminatory serum and CSF metabolites distinguishing DTH and control animals at day 12 when there is the greatest metabolomics separation, variable importance in projection (VIP) scores from the OPLS-DA models were generated. The VIP score summarizes the contribution a variable makes to the model and hence its discriminatory importance—a higher score represents a greater contribution [30]. The top 2 serum and CSF metabolites were chosen to enable an in-depth analysis of their concentrations across animal groups and disease course.

**Fig. 6:**
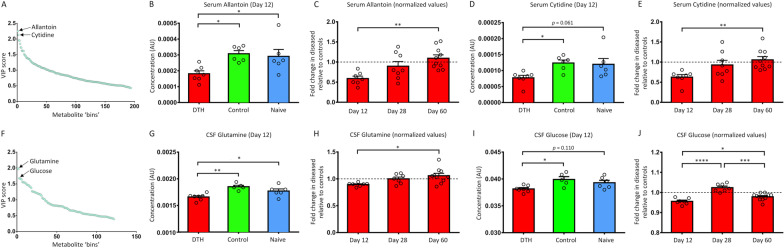
Top discriminatory serum and CSF metabolites. **A** VIP score ranking plot obtained from OPLS-DA modelling of serum CPMG spectral data at day 12, showing that allantoin and cytidine had the highest VIP scores. **B** DTH animals had lower serum concentrations of allantoin compared to control and naïve animals at day 12, indicating that low allantoin was specific for the presence of the DTH lesion. **C** Fold change in allantoin concentrations within DTH animals, normalized to the mean concentrations within controls at each time point (indicated by horizontal dashed line at 1.0), revealing that levels increased with time to that seen in controls. **D** Serum cytidine levels were lower in DTH animals at day 12 when compared to control and naïve animals although the difference between DTH and naïve animals did not reach statistical significance. **E** Normalized data showing that cytidine levels increased with time to that observed in control animals. **F** VIP score ranking plot derived from the CSF OPLS-DA models at day 12, showing that glutamine and glucose had the highest VIP scores. **G** Lower CSF glutamine concentrations were observed in DTH animals at day 12, compared to control and naïve animals. **H** Glutamine concentrations within DTH animals (normalized to controls) increased with time. **I** Glucose concentrations were also decreased in DTH animals although statistical significance was not reached on post hoc comparison between DTH and naïve animals. **J** Normalized glucose levels showing that glucose levels were increased at days 28 and 60, with a peak at day 28. ****p < 0.0001, ***p < 0.001, **p < 0.01, *p < 0.05 by post hoc Tukey test after one-way ANOVA. Data presented as mean ± SEM. *ANOVA* analysis of variance, *AU* arbitrary units, *CPMG* Carr–Purcell–Meiboom–Gill, *DTH* delayed-type hypersensitivity, *OPLS-DA* orthogonal partial-least square discriminant analysis, *SEM* standard error of mean, *VIP* variable importance in projection

The VIP ranking plot obtained from the serum OPLS-DA models is shown in Fig. 6A. The top 2 serum metabolites were confirmed to be allantoin and cytidine, and their contributing spectral bins and VIP scores are detailed in Additional file 5: Table S1.

To demonstrate that altered allantoin and cytidine concentrations were specific for the DTH lesion at day 12, one-way ANOVA of both metabolites across all 3 treatment groups were performed (Fig. 6B, D)**,** revealing that allantoin and cytidine levels were lower in DTH compared to control and naive animals, although the difference in cytidine concentration between DTH and naïve animals did not reach statistical significance (*p* = 0.061).

To explore how allantoin and cytidine concentrations change within DTH animals over disease course, data were first normalized to mean levels in controls at each time point. One-way ANOVA was then performed across time points within DTH animals, revealing that allantoin and cytidine concentrations increased with time back to levels seen in controls (Fig. 6C, E).

The same approach was used to identify the top discriminatory CSF metabolites at day 12. The VIP ranking plot derived from the CSF OPLS-DA models is shown in Fig. 6F. Glutamine and glucose were identified as the top 2 metabolites, and their contributing spectral bins and VIP scores are detailed in Additional file 6: Table S2.

To determine whether the changes in glutamine and glucose levels were attributable to the DTH lesion, one-way ANOVA of both metabolites at day 12 across all 3 treatment groups were performed (Fig. 6G, I). Glutamine concentration was lower in DTH animals compared to control and naïve animals, and while this was also observed for glucose, the difference between DTH and naïve animals did not reach statistical significance (*p* = 0.110).

Glutamine and glucose concentrations within DTH animals were then normalized to controls at each time point to explore their fluxes over the time. One-way ANOVA performed across the time points within DTH animals showed that glutamine concentrations increased with time to levels seen in controls, while glucose concentration was the lowest at day 12 and peaked at day 28 (Fig. 6H, J).

### Correlation of top serum and CSF metabolites with histology

To investigate if the top serum (allantoin and cytidine) and CSF (glutamine and glucose) metabolites can inform on underlying pathological processes, correlations with histological markers were performed. Normalized data (DTH animals relative to controls) were used for correlation analyses as these represented changes attributable to the DTH lesion.

**Fig. 7:**
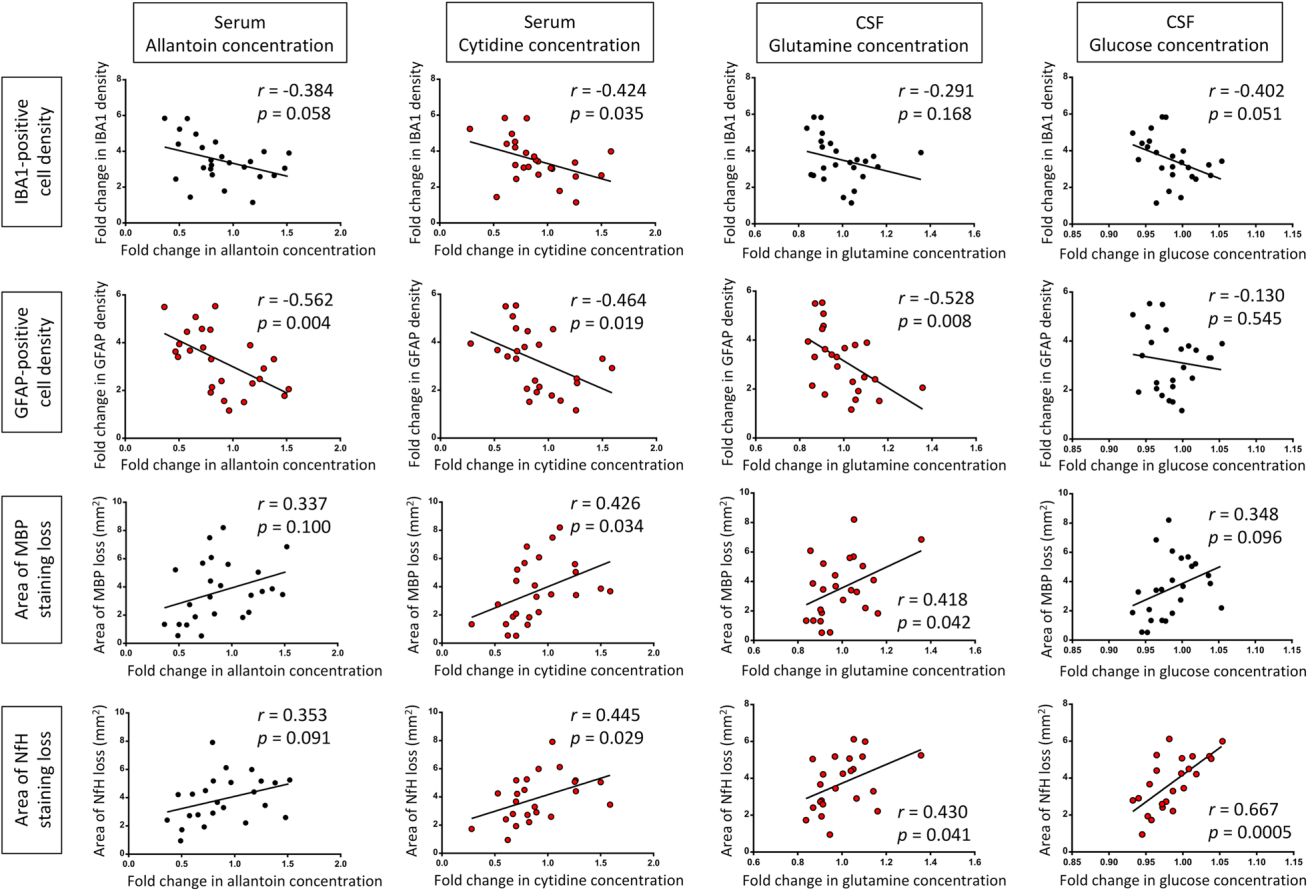
Correlation matrix showing correlation indices between serum allantoin, serum cytidine, CSF glutamine, and CSF glucose with histological markers. Statistically significant correlations are indicated by red symbols. *GFAP* glial fibrillary acidic protein, *IBA1* ionized calcium binding adaptor molecule 1, *MBP* myelin basic protein, *NfH* neurofilament-heavy chain, *r* Pearson correlation coefficient

Serum allantoin concentration correlated inversely with astrocyte cell density (*r* = − 0.562, *p* = 0.004) (Fig. 7). Serum cytidine concentration showed modest correlation with all 4 histological markers—inversely with neuroinflammatory markers (*r* range: − 0.424 to − 0.464) and positively with markers of tissue damage (*r* range: 0.426–0.445). CSF glutamine concentration was inversely correlated with astrocyte cell density (*r* = − 0.528, *p* = 0.008) and positively correlated with area of myelin and axonal loss, albeit modestly (*r* range: 0.418–0.430). Glucose concentration was positively correlated with area of axonal loss, with an *r* of 0.667 (*p* = 0.0005), indicating moderately strong correlation.

## Discussion

The results presented here reveal that: (1) serum and CSF metabolic perturbations from a focal DTH lesion were detectable at all experimental time points, with the greatest perturbations occurring at the earliest time point (i.e. day 12) when the lesion area was least, but the inflammatory response was most intense, (2) the top discriminatory serum and CSF metabolites identified at day 12 were specific for the presence of the DTH lesion; the magnitude of their changes decreased with time to return to the levels observed in controls, and (3) the discriminatory metabolites correlated with histological markers of neuroinflammation and tissue damage; in particular, there were robust correlations for astrogliosis with serum allantoin and CSF glutamine, as well as axonal loss with CSF glucose.

While alteration in the metabolome could be used to distinguish between DTH and control animals at day 12, CSF and serum NfL concentrations could not. Indeed, NfL could only be used to separate the groups at day 28 and this was no longer possible at day 60. It is of note that there were still serum metabolomics differences between DTH and control animals at day 28 and at day 60. Interestingly, CSF and serum NfL levels did not correlate with neuroinflammatory histological markers, but both correlated with myelin loss. Serum NfL alone showed a robust correlation with axonal loss as revealed by NfH staining. The absence of a correlation between CSF NfL levels and NfH-determined histological axonal loss may reflect the topological relationship between the position of the lesion and the CSF sampling point; the lesion being present within the forebrain rather than the spinal cord. Thus, the assumption that NfL enter the blood via the CSF may not be true for all lesions along the neuraxis. Our findings also indicate that, in contrast to the metabolome, NfL is a delayed marker of lesion formation/tissue damage as there is already histological evidence of axonal loss and demyelination at day 12 in the model. The turnover rate and half-life of NfL in humans is poorly understood, but, from these studies, it is now clear that the appearance of NfL occurs later in the pathogenesis of an MS-like lesion. Plausible explanations could be the slow release of NfL from damaged axons into the brain interstitial fluid, and/or the slow transport from there into the CSF via the glymphatics/intramural peri-arterial drainage pathways [34, 35]. We also observed that CSF and serum NfL levels correlated with one another only when elevated within DTH animals, while correlations were not present in control and naïve animals. This finding is consistent with another study investigating plasma and CSF NfL levels in murine models of tauopathy, alpha-synucleinopathy, and β-amyloidosis [36]. Strong correlations (*r* range: 0.86–0.94) were noted in diseased animals in which CSF NfL concentrations of > 20,000 pg/mL were observed. However, in wild-type animals, whereby CSF NfL concentrations were mostly < 1000 pg/mL, the correlation was weaker (*r* = 0.47). These observations suggest that such correlations are present only if the CSF NfL concentration exceeds a high enough threshold, presumably to allow enough to leak into the blood for a corresponding change to be detected.

The OPLS-DA approach enabled us to construct discriminatory models, and in so doing, identify the principal discriminatory metabolites—serum allantoin and cytidine, and CSF glutamine and glucose. Their correlations with histological markers of neuroinflammation and tissue damage lend support for the interrogation of their metabolic pathways to provide inference to the pathophysiological processes underlying DTH lesion formation.

Allantoin is the metabolic end product of purine catabolism in rodents [37, 38]. In humans, a mutation in the gene encoding urate oxidase, which converts uric acid to allantoin, renders the enzyme non-functional [39], thus uric acid is the metabolic end product. It has been noted that uric acid is a potent anti-oxidant in blood and a natural scavenger of peroxynitrite [40], a highly reactive nitrogen species that can cause lipid peroxidation, DNA strand breakage, protein damage via nitration of tyrosine residues, and impair mitochondrial respiration [39, 41]. Indeed, nitrotyrosine (from tyrosine nitration by peroxynitrite) has been demonstrated in brain tissues from MS patients [42]. Experimentally, it has been shown that treatment with uric acid after the onset of clinical signs in the MBP-EAE model improved clinical symptoms and prolonged survival [43], and decreased blood–brain barrier permeability and inducible nitric oxide synthase mRNA expression in the spinal cord [44].

The low allantoin concentrations observed is consistent with other metabolomics studies in MOG-EAE models showing lower plasma and urine allantoin levels as compared to controls [17, 18]. This could be the consequence of low uric acid levels resulting from a consumptive process in which uric acid is utilized to scavenge reactive oxygen/nitrogen species produced by reactive immune/glial cells. Indeed, several clinical studies (including a meta-analysis) have reported lower uric acid levels in MS compared to controls [45], with one study reporting longitudinal reduction in levels within patients [46], however, the chemical structure of uric acid renders it invisible to ^1^H NMR spectroscopy, precluding confirmation of this hypothesis in this study.

Cytidine is a pyrimidine nucleoside required for RNA synthesis and is also a precursor for cytidine triphosphate required for the production of phosphatidylcholine, which is a major component of the neuronal membrane and myelin [47, 48]. A study using the MOG-EAE model as well as the cuprizone model showed that supplementation with cytidine diphosphocholine enhanced remyelination and improved clinical scores [49]. Therefore, the low cytidine concentration observed could be due to: (1) increased incorporation into RNA to meet transcriptional demands during acute neuroinflammation, and/or (2) increased utilization to produce membrane/myelin components after injury, especially when it is known that cytidine can cross the blood–brain barrier [47].

Perturbations in CSF glucose and glutamine (a conditionally essential amino acid) point towards an energy deficient state within the CNS resulting from metabolic decoupling between neurons and glial cells. Recently, we have demonstrated that the concentrations of these 2 metabolites were lower in the CSF of MS patients compared to non-MS controls [11], consistent with the results in other clinical studies [50, 51]. Our finding of reduced CSF glutamine is also consistent with other metabolomics studies in animals showing lower concentrations in MBP-EAE Lewis rats vs. non-immunized controls [14, 15].

During neuroinflammation, low CSF glucose could arise from several deranged metabolic pathways including: (1) increased glucose uptake by neurons as the major fuel substrate for adenosine triphosphate (ATP) production caused by reduced lactate shuttling from astrocytes, (2) increased anaerobic glycolysis in neurons from reduced oxidative phosphorylation due to mitochondrial failure, and (3) increased glucose uptake by glial/immune cells to meet energy demands required to mount an inflammatory response [52, 53]. Reduced CSF glutamine could be a result of: (1) glutaminolysis in glial/immune cells to produce glutamate that is then converted to α-ketoglutarate to be used a substrate in the Krebs cycle for ATP production [54, 55], (2) increased uptake by T-cells and macrophages to support various immune functions including cytokine production [53], and/or (3) disruption of the glutamine–glutamate cycle between astrocytes and neurons [52], although glutamate was not amongst the principal discriminatory metabolites.

In conclusion, we have shown that serum and CSF metabolomics, and indeed selected metabolites, are able to detect early subclinical lesions and that these perturbations normalize with time. This suggests that metabolomics could be explored as potential biomarkers of subclinical disease activity, and dovetails with our recent findings on its application to differentiate clinical relapses from remission [12]. Indeed, metabolomics and NfL levels may be utilized in a complementary manner—metabolomics as an early tool to detect newly developing clinically silent neuroinflammation, with NfL as a latter marker to confirm neuroaxonal injury, providing a holistic surrogate for MS lesions throughout their evolution.

## Supplementary Information

**Additional file 1: Fig. S1** Weight of all animals at the 3 experimental time points. No weight differences were observed between the treatment groups at each time point on two-way ANOVA. *ANOVA: analysis of variance; DTH: delayed-type hypersensitivity*

**Additional file 2: Fig. S2** CSF and serum NfL concentrations in control and naïve animals. No differences in NfL concentrations were observed between the 2 groups in both (A) CSF and (B) serum on two-way ANOVA. *ANOVA: analysis of variance; NfL: neurofilament-light chain*

**Additional file 3: Fig. S3** Correlation between CSF and serum NfL concentrations. (A) In all animals at all time points, (B) stratified by treatment groups at all time points, and (C) within DTH animals stratified by time points. *DTH: delayed-type hypersensitivity; NfL: neurofilament-light chain; r: Pearson correlation coefficient*

**Additional file 4: Fig. S4** Comparison of clean vs. blood-contaminated CSF NMR spectra. (A) 1D-NOESY-presat spectra (chemical shift shown: 0.60 ppm – 4.20 ppm) demonstrating clean CSF (in blue) and blood-contaminated CSF (in red). This revealed aberrant NMR resonances contributed by lipoproteins and macromolecules (e.g. albumin) in blood, resulting in a broad signal with an elevated baseline (boxed region, chemical shift shown: 0.70 ppm – 1.40 ppm). (B) Zoom-in view of this boxed region showed additional resonances arising from the mobile methyl (CH_3_) and methylene (CH_2_) groups within lipoproteins, as well as from the branched chain amino acids (i.e. isoleucine, leucine, and valine). *NMR: nuclear magnetic resonance; NOESY: nuclear overhauser effect spectroscopy; ppm: parts per million*

**Additional file 5: Table S1** The top 2 serum metabolites differentiating DTH and control animals at day 12.

**Additional file 6: Table S2** The top 2 CSF metabolites differentiating DTH and control animals at day 12. *As multiple ‘bins’ are attributable to glucose, only those with a VIP rank of < 10 are shown.

## Data Availability

The datasets used and/or analysed during the current study are available from the corresponding author on reasonable request.

## Abbreviations

ANOVA: Analysis of variance
ATP: Adenosine triphosphate
AU: Arbitrary units
BCG: Bacillus Calmette–Guérin
CFU: Colony forming units
CNS: Central nervous system
CPMG: Carr–Purcell–Meiboom–Gill
CSF: Cerebrospinal fluid
DTH: Delayed-type hypersensitivity
EAE: Experimental autoimmune encephalomyelitis
GFAP: Glial fibrillary acidic protein
IBA1: Ionized calcium binding adaptor molecule 1
MBP: Myelin basic protein
MRI: Magnetic resonance imaging
MS: Multiple sclerosis
M.tb: *Mycobacterium tuberculosis*
NfH: Neurofilament-heavy chain
NfL: Neurofilament-light chain
NMR: Nuclear magnetic resonance
NOESY: Nuclear overhauser effect spectroscopy
OPLS-DA: Orthogonal partial-least square discriminant analysis
PBS: Phosphate-buffered saline
*r*: Pearson correlation coefficient
SD: Standard deviation
SEM: Standard error of mean
VIP: Variable importance in projection
