# Supplementary figures and images for: Metabolomics detects clinically silent neuroinflammatory lesions earlier than neurofilament-light chain in a focal multiple sclerosis animal model
Source: J Neuroinflammation. 2022 Oct 9;19:252. doi: 10.1186/s12974-022-02614-8 (PMC9549622; doi:10.1186/s12974-022-02614-8)

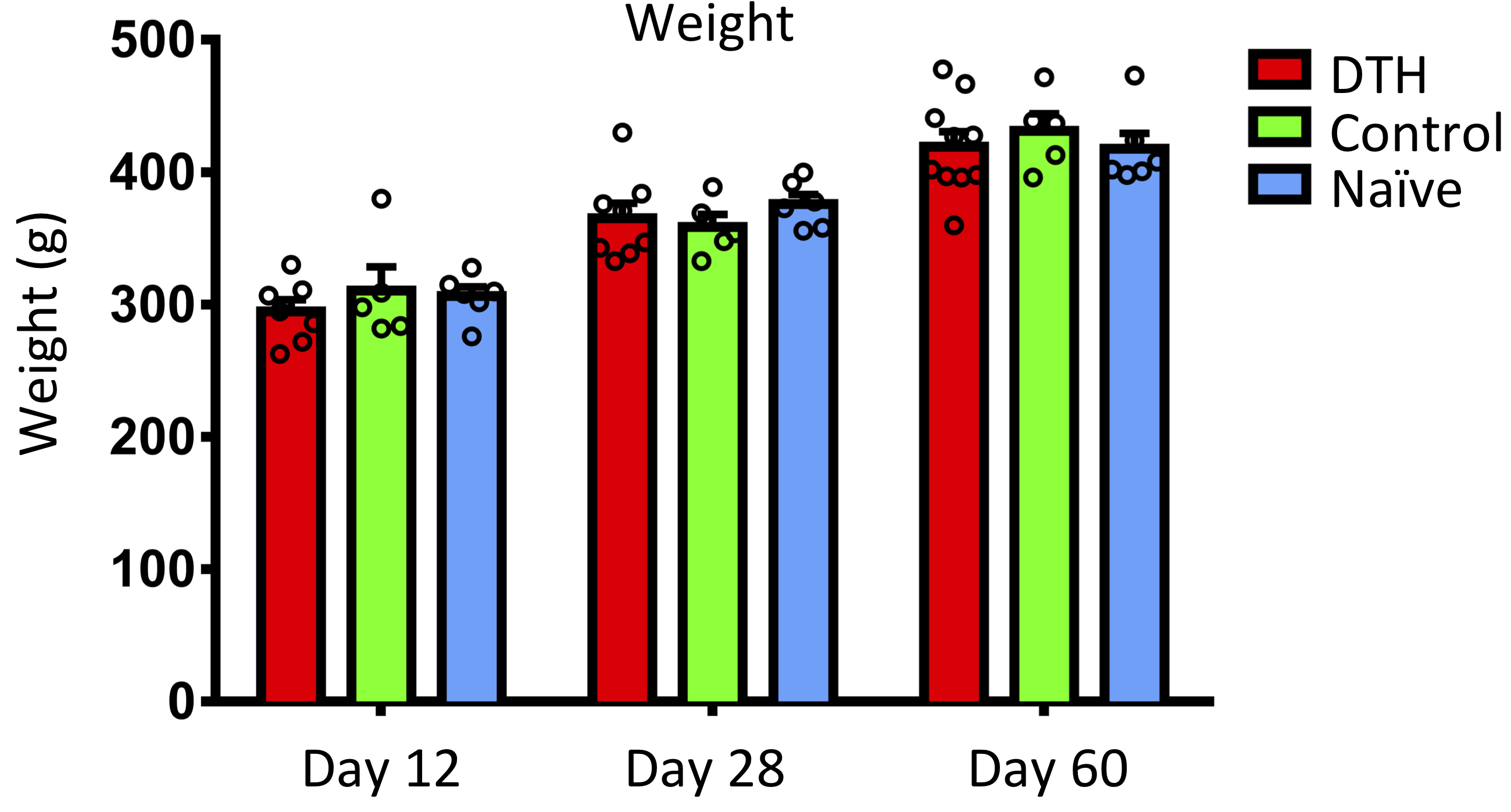

Supplement: Supplementary file 1 — Additional file 1: Fig. S1 Weight of all animals at the 3 experimental time points. No weight differences were observed between the treatment groups at each time point on two-way ANOVA. ANOVA: analysis of variance; DTH: delayed-type hypersensitivity [file 12974_2022_2614_MOESM1_ESM.tiff]

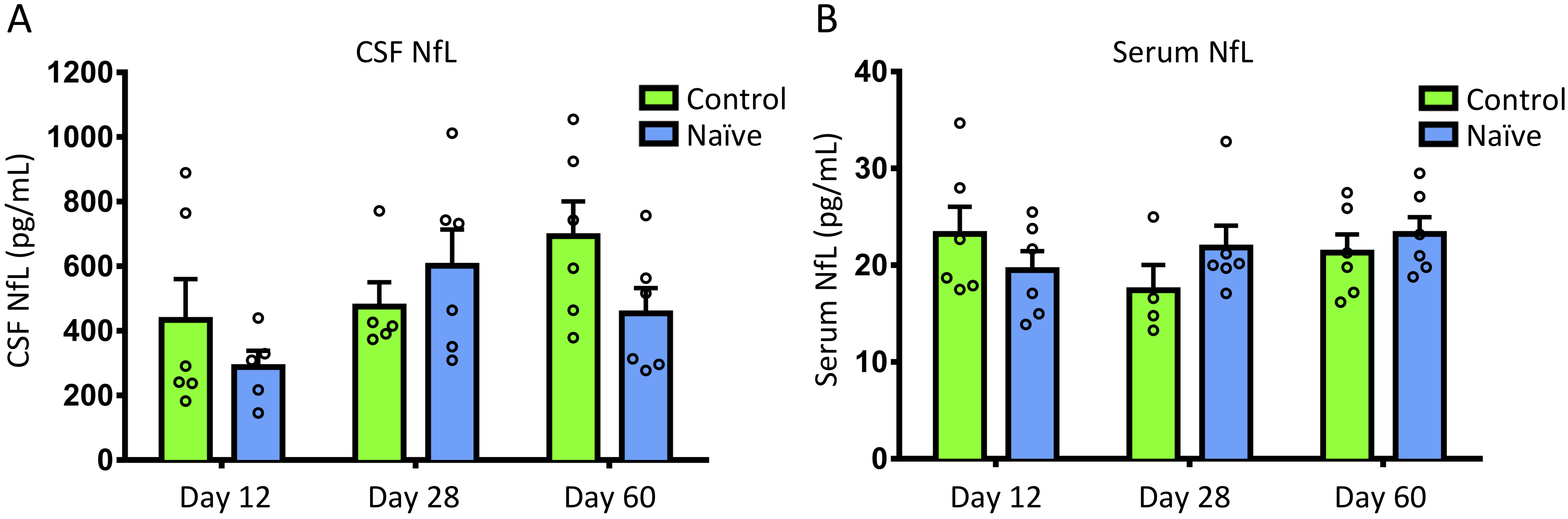

Supplement: Supplementary file 2 — Additional file 2: Fig. S2 CSF and serum NfL concentrations in control and naïve animals. No differences in NfL concentrations were observed between the 2 groups in both (A) CSF and (B) serum on two-way ANOVA. ANOVA: analysis of variance; NfL: neurofilament-light chain [file 12974_2022_2614_MOESM2_ESM.tiff]

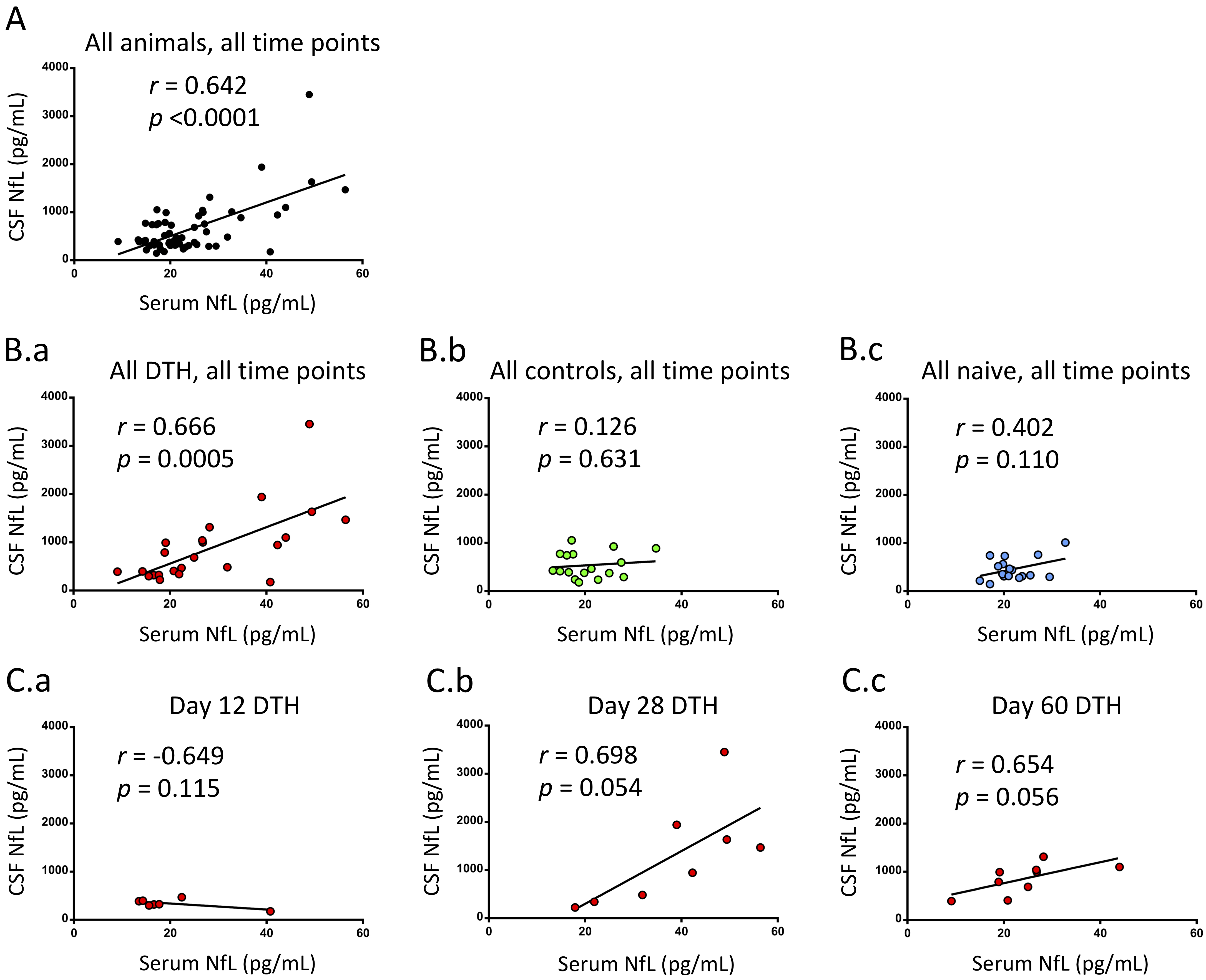

Supplement: Supplementary file 3 — Additional file 3: Fig. S3 Correlation between CSF and serum NfL concentrations. (A) In all animals at all time points, (B) stratified by treatment groups at all time points, and (C) within DTH animals stratified by time points. DTH: delayed-type hypersensitivity; NfL: neurofilament-light chain; r: Pearson correlation coefficient [file 12974_2022_2614_MOESM3_ESM.tiff]

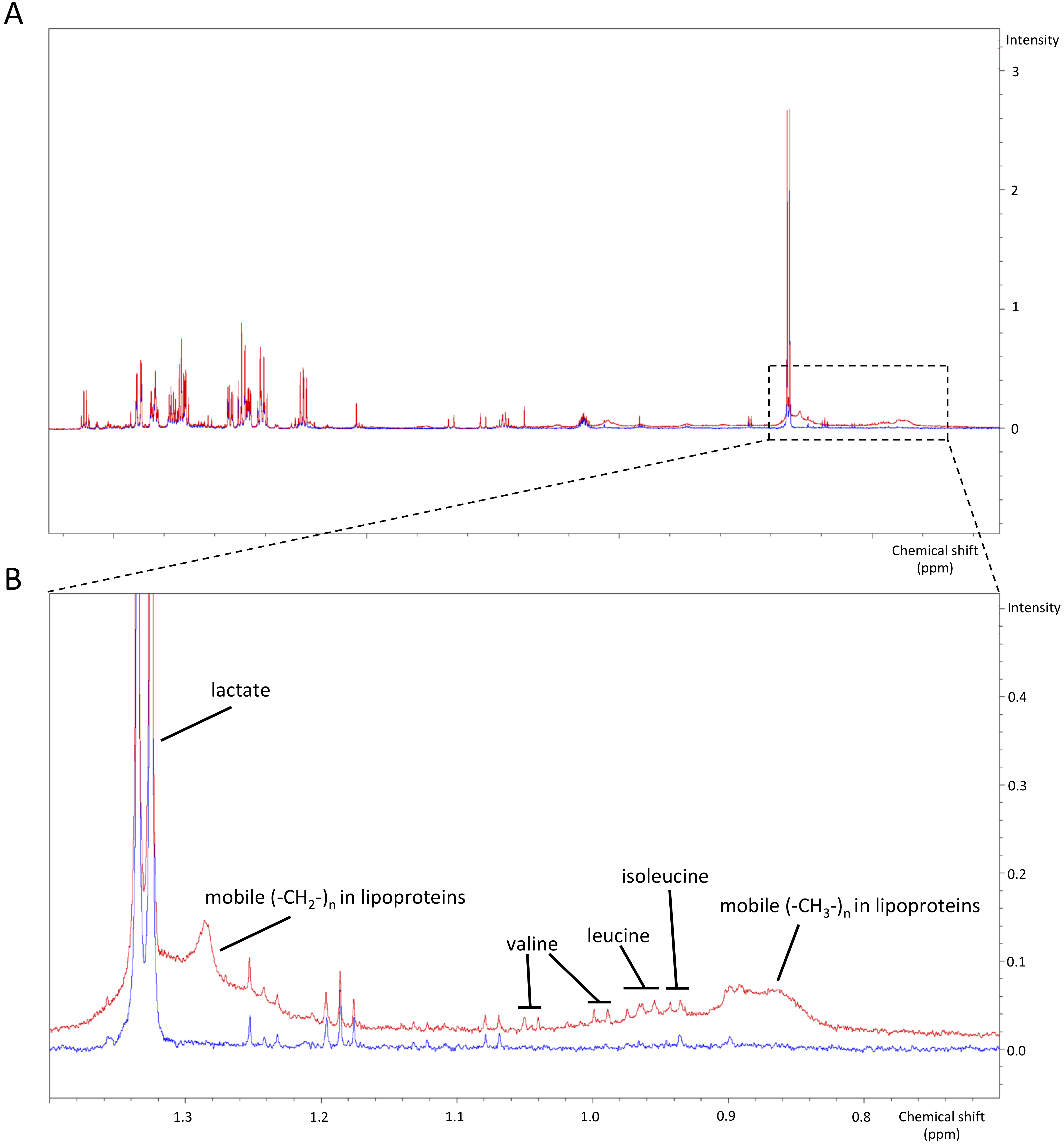

Supplement: Supplementary file 4 — Additional file 4: Fig. S4 Comparison of clean vs. blood-contaminated CSF NMR spectra. (A) 1D-NOESY-presat spectra (chemical shift shown: 0.60 ppm – 4.20 ppm) demonstrating clean CSF (in blue) and blood-contaminated CSF (in red). This revealed aberrant NMR resonances contributed by lipoproteins and macromolecules (e.g. albumin) in blood, resulting in a broad signal with an elevated baseline (boxed region, chemical shift shown: 0.70 ppm – 1.40 ppm). (B) Zoom-in view of this boxed region showed additional resonances arising from the mobile methyl (CH3) and methylene (CH2) groups within lipoproteins, as well as from the branched chain amino acids (i.e. isoleucine, leucine, and valine). NMR: nuclear magnetic resonance; NOESY: nuclear overhauser effect spectroscopy; ppm: parts per million [file 12974_2022_2614_MOESM4_ESM.tiff]
