# Supplementary material for: Metabolomics detects clinically silent neuroinflammatory lesions earlier than neurofilament-light chain in a focal multiple sclerosis animal model
Source: J Neuroinflammation. 2022 Oct 9;19:252. doi: 10.1186/s12974-022-02614-8 (PMC9549622; doi:10.1186/s12974-022-02614-8)
Supplement: Supplementary file 5 — Additional file 5: Table S1 The top 2 serum metabolites differentiating DTH and control animals at day 12. [file 12974_2022_2614_MOESM5_ESM.docx]

| **Top 2 discriminatory metabolites** | **Chemical shift of contributing spectral ‘bins’ (VIP score, VIP rank)** |
| --- | --- |
| Allantoin | 5.38….5.40 ppm (2.25, 1) |
| Cytidine | 6.04….6.06 ppm (2.13, 2)  6.06….6.08 ppm (1.84, 5) |

**Table S1** The top 2 serum metabolites differentiating DTH and control animals at day 12.

ppm: parts per million; VIP: variable importance in projection
