# Supplementary material for: Metabolomics detects clinically silent neuroinflammatory lesions earlier than neurofilament-light chain in a focal multiple sclerosis animal model
Source: J Neuroinflammation. 2022 Oct 9;19:252. doi: 10.1186/s12974-022-02614-8 (PMC9549622; doi:10.1186/s12974-022-02614-8)
Supplement: Supplementary file 6 — Additional file 6: Table S2 The top 2 CSF metabolites differentiating DTH and control animals at day 12. *As multiple ‘bins’ are attributable to glucose, only those with a VIP rank of < 10 are shown. [file 12974_2022_2614_MOESM6_ESM.docx]

| **Top 2 discriminatory metabolites** | **Chemical shift of contributing spectral ‘bins’ (VIP score, VIP rank)** |
| --- | --- |
| Glutamine | 2.48….2.50 ppm (1.99, 1)  2.40….2.42 ppm (1.96, 2) |
| Glucose* | 3.70….3.72 ppm (1.68, 3)  3.74….3.76 ppm (1.66, 5)  3.44….3.46 ppm (1.64, 6)  3.82…3.84 ppm (1.55, 8) |

**Table S2** The top 2 CSF metabolites differentiating DTH and control animals at day 12. *As multiple ‘bins’ are attributable to glucose, only those with a VIP rank of <10 are shown.

DTH: delayed-type hypersensitivity; ppm: parts per million; VIP: variable importance in projection
